# Antiviral Therapy for Varicella Zoster Virus (VZV) and Herpes Simplex Virus (HSV)-Induced Anterior Uveitis: A Systematic Review and Meta-Analysis

**DOI:** 10.3389/fmed.2021.686427

**Published:** 2021-07-02

**Authors:** Ilaria Testi, Kanika Aggarwal, Nishant Jaiswal, Neha Dahiya, Zheng Xian Thng, Aniruddha Agarwal, Alka Ahuja, Mona Duggal, Ankita Kankaria, Su Ling Ho, Soon-Paik Chee, Mark Westcott, Carlos Pavesio, Rupesh Agrawal, Vishali Gupta

**Affiliations:** ^1^Department of Uveitis, Moorfields Eye Hospital, NHS Foundation Trust, London, United Kingdom; ^2^Advanced Eye Centre, Post-Graduate Institute of Medical Education and Research (PGIMER), Chandigarh, India; ^3^Department of Telemedicine, Postgraduate Institute of Medical Education and Research, Chandigarh, India; ^4^National Healthcare Group Eye Institute, Tan Tock Seng Hospital, Singapore, Singapore; ^5^Department of Community and Family Medicine, All India Institute of Medical Sciences (AIIMS), Bathinda, India; ^6^Singapore Eye Research Institute, Singapore, Singapore; ^7^Singapore National Eye Centre, Singapore, Singapore; ^8^Department of Ophthalmology, National University of Singapore, Singapore, Singapore; ^9^Duke National University of Singapore Medical School, Singapore, Singapore; ^10^Lee Kong Chian School of Medicine, Nanyang Technological University, Singapore, Singapore

**Keywords:** viral anterior uveitis, iritis, herpes simple virus, varicella zoster virus, herpes zoster ophtalmicus, antiviral therapy, acyclovir, valaciclovir

## Abstract

**Topic:** Herpes simplex virus (HSV) and varicella zoster virus (VZV) are the most common ocular pathogens associated with infectious anterior uveitis. Currently, there are a number of antiviral agents administered to treat viral anterior uveitis (VAU). However, there is no consensus or guidelines about the most appropriate approach leading for the best treatment outcomes with fewer ocular complications.

**Clinical Relevance:** To perform a systematic review and meta-analysis of the efficacy of different antiviral therapies in the management of anterior uveitis secondary to HSV and VZV.

**Methods:** We searched PubMed, Web of Science, CINAHL, OVID, and Embase up to January 2020. Randomized trials, non-randomized intervention studies, controlled before and after studies and observational studies assessing the effect of oral and or topical treatments for VAU were considered. Data extraction and analysis with evaluation of the risk of bias in the included trials were performed.

**Results:** Oral acyclovir demonstrated a statistically significant good treatment outcome in the management of VZV anterior uveitis (vs. placebo) (OR 0.26, 95% CI 0.11–0.59), but did not have similar effect in HSV anterior uveitis (OR 0.47, 95% CI 0.15–1.50). In the treatment of VZV anterior uveitis, there was significant superiority of oral acyclovir−7 day course—over topical acyclovir (OR 4.17, 95% CI 1.28–13.52). Whereas, there was no significant superiority of one of the following treatment regimens over the others: topical acyclovir over topical corticosteroids (OR 1.86, 95% CI 0.67–5.17), and oral acyclovir−7 day course—over oral acyclovir−14 day course—(OR 0.21, 95% CI 0.01–4.50) or oral valaciclovir (OR 1.40, 95% CI 0.48–4.07).

**Conclusion:** Treatment of HSV and VZV anterior uveitis is currently based on individual experiences and limited literature, largely due to weak clinical trial evidence in this regard. Our results highlight the existence of a substantial gap in our evidence base. This finding might contribute to future research studies to ascertain the role of different antiviral therapies in the treatment of VAU.

**Systematic Review Registration:** PROSPERO registration number: CRD420202 00404.

## Background

Viral anterior uveitis (VAU) is the most common form of infectious uveitis, accounting for more than 10% of all cases of anterior uveitis (1). Different members of the herpes virus family, including herpes simplex virus (HSV) and varicella zoster virus (VZV), are considered for the differential diagnosis when a viral etiology is suspected.

The diagnosis is mostly clinical (2–4). The presence of herpetic dermatitis, including vesicles occurring at the border of the eyelids and the zoster vesicular rash, or dendritic keratitis can provide a strong corroborative evidence for the diagnosis, but these findings are often absent. The presumptive diagnosis is thus based on ocular features, including unilateral involvement, reduced corneal sensation, keratic precipitates, iris atrophy and rise in intraocular pressure, with a course of the disease that is commonly recurrent (2–4). However, considering the overlap between clinical features suggestive of HSV and VZV anterior uveitis, once a viral etiology is suspected, polymerase chain reaction (PCR)-based analysis of aqueous humor sample may be used by the clinicians for confirming the diagnosis (5–8). The detection of the pathogen allows a more targeted therapy with the aim of limiting further spread of the virus and avoiding secondary tissue damage.

Treatment management of VAU is tailored to the virus and to the clinical immune response that the pathogen induces (9). Hence, therapeutic regimen usually includes antiviral medications (systemic and/or topical) in combination with topical inflammatory agents, commonly corticosteroids (1–3, 10–23). Cycloplegics and/or intraocular pressure lowering eye drops are the adjunctive therapy based on individual patient's clinical condition. Different antiviral agents for varying duration have been used in the treatment of VAU, including acyclovir and valacyclovir (1–3, 10–23).

To gain insight into the effectiveness of different therapeutic strategies and to propose the most appropriate treatment strategy for VAU, high-quality data collection or a well-designed randomized controlled trial is warranted. The objective of this systematic review and meta-analysis is hence to assess and compare the effectiveness of antiviral therapies, administered alone or in combination with other agents, in the management of anterior uveitis secondary to HSV and VZV. Cytomegalovirus (CMV) has not been considered for the purpose of this analysis.

## Methods

The study protocol investigating the role of antiviral therapy for VZV and HSV induced anterior uveitis can be found at PROSPERO, international database of prospectively registered systematic reviews (registration number: CRD42020200404). The study was performed in accordance to the PRISMA (Preferred Reporting Items for Systematic Reviews and Meta-Analyses) guidelines. The PRISMA checklist is provided in Supplementary Data Sheet 1.

The inclusion criteria were:

### Participants

Acute or chronic cases of viral anterior uveitis, caused by HSV or VZV, diagnosed on the basis of clinical criteria with or without confirmation by Goldmann-Witmer coefficient (GWC) or polymerase chain reaction (PCR) were included. Other inclusion criteria were: any age group (children and adults), either gender, any race and ethnicity, irrespective of the immune status, who received antiviral medications, either topical or systemic, including acyclovir, valacyclovir, famciclovir, ganciclovir, and valganciclovir.

### Types of Studies

a. Randomized trials which assess the effect of oral and or topical treatments for viral anterior uveitis.
b. Non-randomized intervention studies and controlled before-after studies in addition to observational studies (including prospective and retrospective cohort and case-control studies, cross-sectional studies).
c. Studies that include subsets of relevant participants if the data for the relevant subsets are reported separately (in such cases, we planned to include only the data for the relevant subsets).

Case reports, letter to editors (not reporting cases), narrative reviews, and correspondence (such as editorials) were excluded.

### Types of Outcome Measure

#### Primary Outcome Measure

Number of patients with treatment failure as per the criteria defined in the included studies such as persistence or worsening of ocular inflammation, development of ocular inflammation, development of viral epithelial keratitis or epithelial defect, persistent rise in intraocular pressure despite maximally tolerated medical therapy, discontinuation of treatment due to an adverse reaction and voluntary withdrawal from study.

#### Secondary Outcome Measures

- Time-to-quiescence, defined as the duration from the presentation/recurrence of anterior uveitis to the time when zero cells were noted in the anterior chamber
- Time-to-recurrence, defined as the duration between achieving quiescence and the next episode
- Change in number of uveitis attacks before and after therapy, defined as mean number of uveitis recurrence/month of follow-up/patient
- Reduction of anti-glaucoma medications and anti-inflammatory eye drops, defined as reduction in the number of intraocular pressure-lowering medications and in the frequency of steroid eye drops applications, respectively.

### Search Methods for Identification of Studies

A systematic literature search of electronic databases i.e., PubMed, Web of Science, CINAHL, OVID, and Embase was performed by 2 independent reviewers. The searches included literature up to 27th August 2020. Publications in English language, or those which had English language translation were included in the analysis. The search strategy is provided in Supplementary Data Sheet 2.

### Data Collection and Analysis

#### Selection of Studies

**Figure 1:**
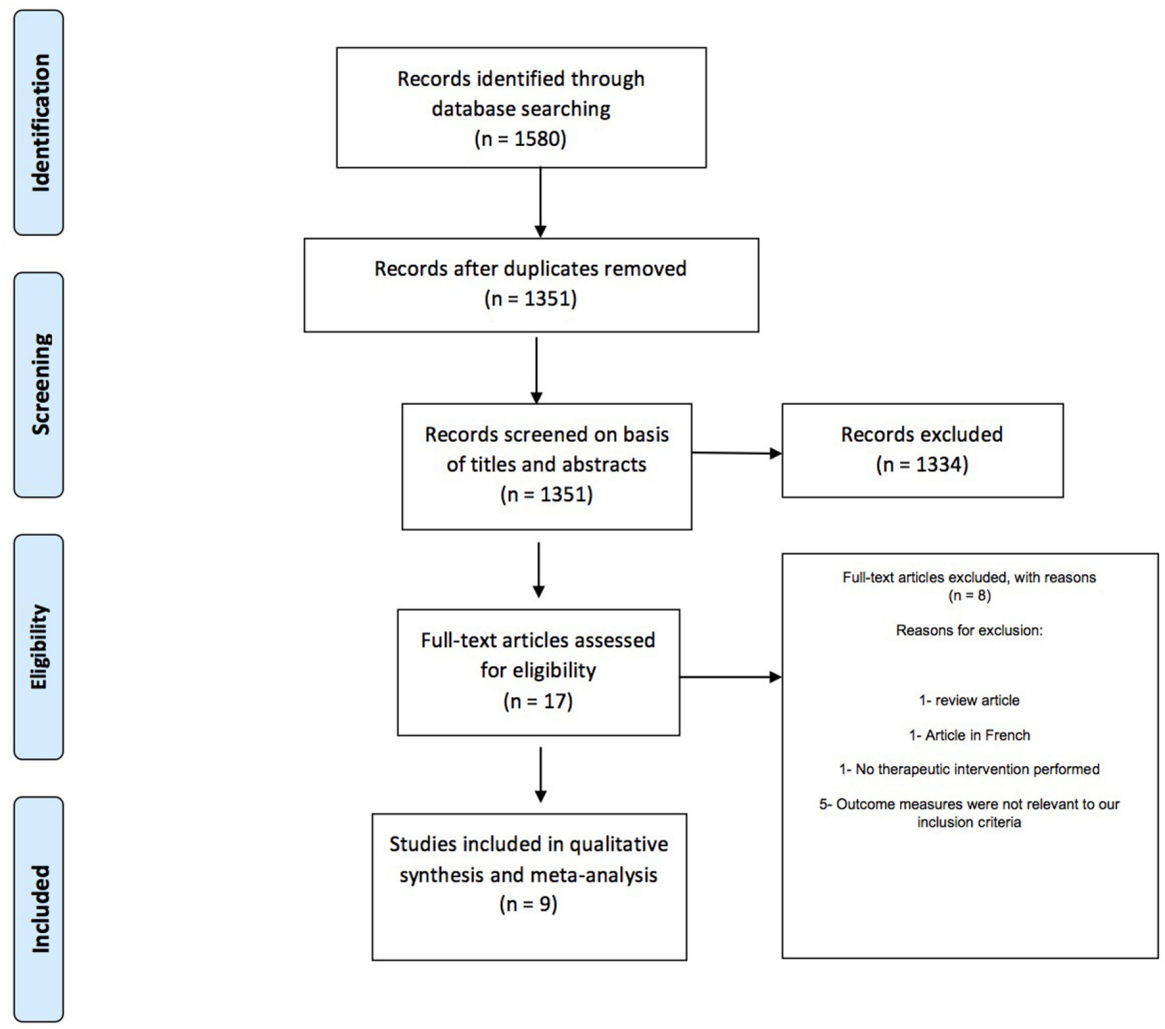
PRISMA flowchart showing study screening and selection process.

The duplicate articles were removed and screening of titles and abstracts was performed by two independent reviewers (IT and VG). Full text screening was done for articles which were not excluded and manuscripts which fit the inclusion criteria were used for data extraction. Any discrepancies were resolved through discussion with a third reviewer (RA) who acted as an arbiter. The screening and selection process has been described in a PRISMA flowchart (Figure 1).

#### Data Extraction

Two reviewers (IT and KA; both fellowship trained uveitis specialists) extracted the data independently on pre-piloted structured forms related to study setting, study design, demographic details of patients, etiology of viral anterior uveitis, various interventions or treatment/s given, duration of treatment, outcomes, adverse reactions to interventions and recurrences of disease. Discrepancies if any, were settled after discussion with a third reviewer (NJ).

### Assessment of Risk of Bias in Included Studies

The risk of bias in each included study was assessed using Cochrane's “Risk of bias” tool (Higgins 2011). The tool assesses the biases introduced on the basis of various domains such as inadequacies in random sequence generation, allocation concealment, blinding of participants and study personnel, blinding of outcome assessments, reporting of incomplete outcome data and selective reporting. The review authors judged each of these criteria to have a low, unclear or high risk of bias.

### Measures of Treatment Effect

For dichotomous data, we used odds ratios (OR) and presented these with 95% confidence intervals (CIs). We used Revman 5.4 and Stata 14 software for meta-analysis.

### Assessment of Heterogeneity

Heterogeneity was judged from both clinical and statistical perspectives. Clinical heterogeneity was assessed on the basis of the similarities of study participants, intervention protocols and outcome measures. The evaluation of statistical heterogeneity included visual inspection, and consideration of the Chi^2^ test and the I^2^ statistic. The following interpretation of I^2^ was applied:

- 0–40%: might not be important;
- 30–60%: may represent moderate heterogeneity;
- 50–90%: may represent substantial heterogeneity;
- 75–100%: considerable heterogeneity.

### Assessment of Reporting Bias

We were unable to construct funnel plots (plotting trial effects against inverse standard errors of effects) to assess for reporting biases as planned, as <10 studies were available for all outcomes included in the meta-analysis.

### Data Synthesis

Data was pooled from similar studies using inverse-variance fixed-effects method. Wherever it was inappropriate to pool data and meta-analysis was not possible, we presented the data in tables for illustrative purposes and a narrative synthesis was done.

## Results

### Results of the Research

Our literature search retrieved a total of 1,580 articles out of which 229 articles were removed as duplicates (Figure 1). The titles and abstracts of the remaining 1,351 articles were screened and irrelevant articles were excluded. Full text screening was done for 17 studies which were assessed on the basis of our inclusion criteria. Eight studies were excluded for being unable to fulfill the inclusion criteria (2, 11, 12, 21, 24–27), and the remaining 9 studies were included in this systematic review (13–20, 23). The characteristics of the included studies are listed in Table 1 (study details and population demographics) and Table 2 (diagnosis, intervention, outcome).

**Table 1:** Study details and population demographics.

**S/N**	**References**	**Study design**	**Origin**	**Sample size**	**No. of patients who received antiviral treatment**	**Type of antiviral treatment**	**Control group**	**No. of control**	**Age (years); mean**	**Male gender (%)**
1	The Herpetic Eye Disease Study Group (20)	Multicentre, controlled clinical trial	USA	50	22	Oral Acyclovir	Placebo	28	53	38%
2	Wilhelmus et al. (23)	Clinical trial	USA	260	73	Oral acyclovir	Placebo; topical corticosteroids	187 (49 + 138)	Not reported	Not reported
3	Marsh and Cooper (18)	Double-masked, randomized trial	UK	83	57	Topical acyclovir	Topical corticosteroids	26	Not reported	Not reported
4	McGill and Chapmen (19)	Controlled trial	UK	40	20	Topical acyclovir	Topical corticosteroids	20	71	33.3%
5	Cobo et al. (13)	Prospective, longitudinal, randomized, double-masked, placebo-controlled trial	USA	71	36	Oral acyclovir	Placebo	35	Not reported	53.5%
6	Colin et al. (14)	Multicentre, randomized, double-masked study	France	110	110 (54 + 56)	Oral acyclovir (54); oral valaciclovir (56)	Not reported	Not reported	62 in the acyclovir treated group and 58 in the valaciclovir treated group	42.5%
7	Harding and Porter (15)	Placebo controlled trial	UK	42	23	Oral acyclovir	Placebo	19	62.1 in the acyclovir treated group and 70.6 in the placebo group	32.6%
8	Hoang-Xuan et al. (16)	Bicentric, prospective, randomized, double- masked trial	France, Switzerland	86	86 (41 + 45)	Oral acyclovir 7 day course (41); oral acyclovir 14 day course (45)	Not reported	Not reported	50.5 in 7 day course group and 56.6 in 14 day course group	46.5%
9	Neoh et al. (17)	Multicentre, open randomized trial	UK	57	57 (26 + 31)	Topical acyclovir (26); oral acyclovir (31)	Not reported	Not reported	64.6	Not reported

**Table 2:** Diagnosis, intervention, outcome.

**S/N**	**References**	**Diagnosis**				**Intervention**		**Outcome**	
		**Etiology**	**Diagnosis**	**Ocular manifestations**	**Local/systemic investigations**	**Patients**	**Controls**	**Criteria for treatment failure**	**Treatment failure**
**1**	The Herpetic Eye Disease Study Group (20)	Presumed HSV	Presence of at least one of the following: (1) history consistent with previous ocular HSV disease, (2) presence of concomitant stromal keratitis consistent with HSV as the cause, or (3) the presence of serum antibodies to HSV in the absence of other identifiable causes of iridocyclitis	Iridocyclitis; active non-necrotizing stromal keratitis; intraocular pressure 30 mm Hg or more; no epithelial keratitis	Serum antibodies to HSV	Oral acyclovir, 400 mg, 5 times daily for 10 weeks + topical trifluridine for 10 weeks + prednisolone phosphate 1% for 10 weeks	Placebo capsules containing 218 mg lactose + topical trifluridine for 10 weeks + prednisolone phosphate 1% for 10 weeks	Increase in severity of stromal keratitis defined as development of a new zone of non-necrotizing or necrotizing stromal keratitis 15 mm^2^ or more in area or increase in total area of previously present non-necrotizing stromal keratitis 7.5 mm^2^ or more; persistent stromal keratitis with <10% reduction in the area of non necrotizing or necrotizing stromal keratitis and no improvement or worsening in all of the ancillary signs by 2 weeks after entry into the trial or over any 3 consecutive weeks; increase in severity of iridocyclitis, defined as a 2-step or greater increase in cells in the anterior chamber; persistent iridocyclitis of 3+ cells or more for 2 consecutive weeks; decrease in visual acuity of 4 or more lines on the modified Bailey-Lovie charts; development of herpes simplex virus (HSV) epithelial keratitis or an epithelial defect more than 1.0 mm in length; intraocular pressure more than 35 mmHg for at least 1 week despite maximally tolerated medical therapy; development of new active ocular HSV disease when off treatment; development of an adverse reaction attributable to trial medications; use of a topical or systemic corticosteroid or antiviral agent other than the trial medications; patient decision to withdraw	50% in oral acyclovir treated group; 67.9% in the placebo treated group
**2**	Wilhelmus et al. (23)	Presumed HSV	Non-necrotising or necrotising stromal keratitis and/or iridocyclitis attributable to HSV on the basis of clinical findings, without active epithelial keratitis or epithelial defect	Active stromal keratitis; iridocyclitis; no epithelial disease	Not reported	Oral acyclovir two 200 mg capsules, 5 times daily for 10 weeks + prednisolone phosphate 1% 8 times daily for 1week, then 6 times daily for 1 week, 4 times daily for 1 week, twice daily for 1 week, then once daily for the fifth week; at the sixth week, prednisolone phosphate 0.125% used 4 time daily for 1 week, then twice daily for the next week, then once daily for the final 3 weeks + topical trifluridine 4 times daily for 3 weeks, then twice daily for 7 weeks	Prednisolone phosphate 1% 8 times daily for 1week, then 6 times daily for 1 week, 4 times daily for 1 week, twice daily for 1 week, then once daily for the fifth week; at the sixth week, prednisolone phosphate 0.125% used 4 time daily for 1 week, then twice daily for the next week, then once daily for the final 3 weeks + topical trifluridine 4 times daily for 3 weeks, then twice daily for 7 weeks; or placebo eye drops - dose and frequency as per prednisolone phosphate regimen - + topical trifluridine 4 times daily for 3 weeks, then twice daily for 7 weeks	Occurrence of HSV epithelial keratitis	2.7% in topical corticosteroids and oral acyclovir treated group; 6.5% in topical corticosteroids group; 2.0% in topical placebo treated group
**3**	Marsh and Cooper (18)	Presumed VZV	Skin lesions of herpes zoster ophthalmicus	Episcleritis; scleritis; keratitis: nummular, disciform, sclero; corneal oedema; iritis	Not reported	Acyclovir ointment 3% ± dexamethasone 0.1%	Placebo ointment + dexamethasone 0.1%	Failure to control intraocular inflammation	70% in the topical acyclovir treated group; 53.8% in topical corticosteroids treated group; 40.7% in topical acyclovir and topical corticosteroids treated group
**4**	McGill and Chapmen (19)	Presumed VZV	Based on clinical ground, backed up by viral isolation when skin vesicles were stll presnt	Epithelial lesion; stromal lesion; uveitis; scleritis	PCR on skin lesions when skin vesicles present	Acyclovir ointment 5 times a day	Betamethasone 0.1% 5 times a day	Recurrence of ocular involvement	5.9% in topical acyclovir treated group; 5.3% in topical corticosteroids treated group
**5**	Cobo et al. (13)	Presumed VZV	Skin lesions of herpes zoster ophthalmicus	Episcleritis; scleritis; dendritiform keratitis; stromal keratitis; corneal scarring/vascularization; anterior uveitis; keratic precipitates; iris atrophy	Not reported	Oral acyclovir 200 mg 3 capsule 5 times daily for 10 days	Placebo 3 capsule 5 times daily for 10 days	Progression of dermatologic or ocular disease during the acute treatment phase	16.6% in oral acyclovir treated group; 51.4% in placebo group
**6**	Colin et al. (14)	Presumed VZV	Skin lesions of herpes zoster ophthalmicus	Episcleritis; superficial keratitis; dendritic ulcer; stromal keratitis; uveitis; elevated intraocular pressure	Not reported	Valaciclovir 1,000 mg 3 times daily for 7 days or acyclovir 800 mg 5 times daily for 7 days	Not reported	Development Of Anterior Uveitis As Intraocular Complication	17% in oral acyclovir treated group; 13% in oral valaciclovir treated group
**7**	Harding and Porter (15)	Presumed VZV	Skin lesions of herpes zoster ophthalmicus	Sclerokeratitis; stromal keratiris; aneterior uveitis	Not reported	acyclovir 800 mg 5 times daily	Placebo capsule 5 times daily	Development of anterior uveitis as intraocular complications	30.4% in oral acyclovir treated group; 52.6% in placebo group
**8**	Hoang-Xuan et al. (16)	Presumed VZV	Skin lesions of herpes zoster ophthalmicus	Episcleritis; superficial keratitis; corneal stromal oedema; anterior stromal infiltrates; anterior uveitis	Not reported	acyclovir 800 mg 5 times daily for 7 days or for 14 days	Not reported	Development of anterior uveitis as intraocular complications	0% in 7 day course group; 4.4% in 14 day course group
**9**	Neoh et al. (17)	Presumed VZV	Skin lesions of herpes zoster ophthalmicus	Episcleritis; sclerokeratitis; keratitis; anterior uveitis	Not reported	oral acyclovir 800 mg 5 times daily for 7 days or acyclovir ointment for 7 days	Not reported	Development of anterior uveitis as intraocular complications	19.3% in oral acyclovir treated group; 50% in topical acyclovir treated group

### Risk of Bias in Included Studies

**Figure 2:**
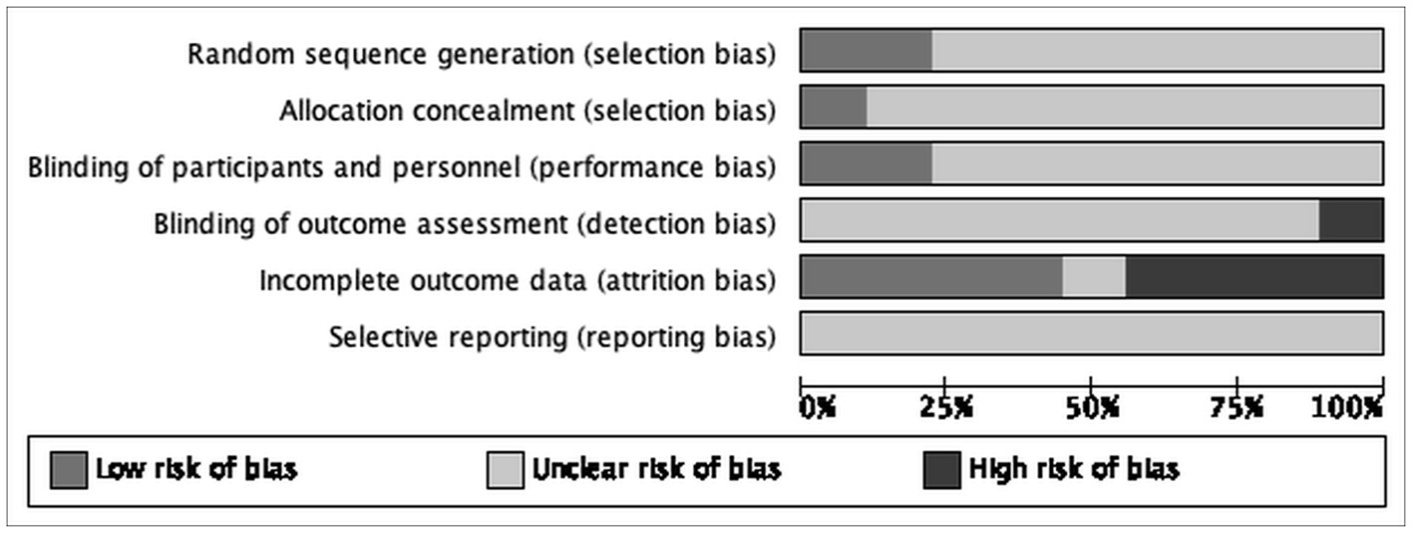
Risk of bias graph: review authors' judgements about each “Risk of bias” item presented as percentages across all included study.

**Figure 3:**
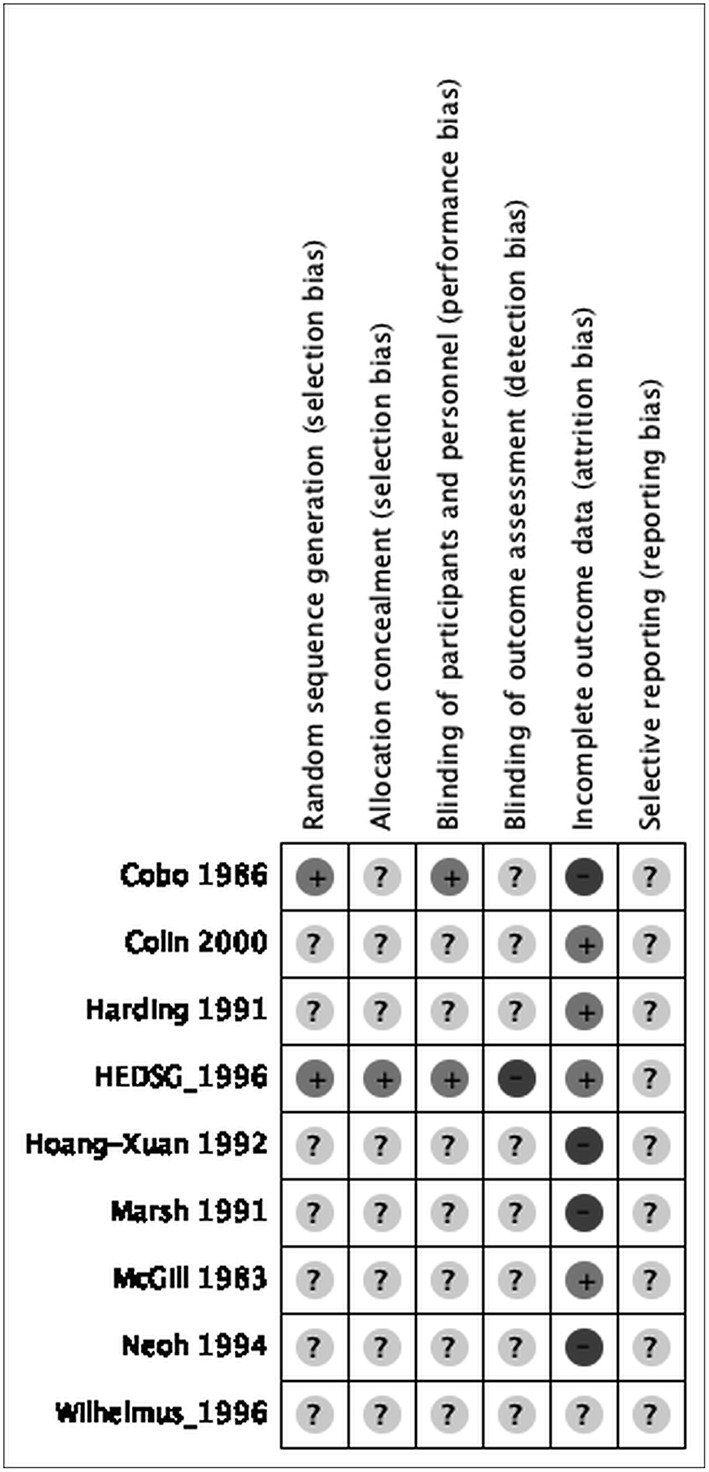
Risk of bias summary: review authors' judgements about each “Risk of bias” item for each included study.

The risk of bias for various domains has been explained in Figures 2, 3 and the section below.

### Allocation

#### Random Sequence Generation (Selection Bias)

We assessed two studies as at low risk of selection bias due to stratified randomization used as details describing a satisfactory method were provided (13, 20). We judged the remaining seven as at unclear risk of bias for this domain as they did not mention the method of randomization (14–19, 23).

#### Allocation Concealment (Selection Bias)

We evaluated one study to be at low risk of bias for this domain as studies concealed the allocations satisfactorily (20). We rated the remaining eight studies to have an unclear risk of bias as they did not report on allocation concealment (13–19, 23).

### Blinding

#### Blinding of Participants and Personnel (Performance Bias)

We judged seven studies to be at unclear risk of performance bias as blinding of participants and personnel was not mentioned (14–19, 23). We rated the remaining two studies at low risk of performance bias as studies concealed the blinding of participants and personnel satisfactorily (13, 20).

#### Blinding of Outcome Assessment (Detection Bias)

We rated one study at high risk of bias as it was not completely blinded (20). We rated the remaining eight studies to be at unclear risk of detection bias as they did not mention blinding (13–19, 23).

### Incomplete Outcome Data

We judged three studies with attrition of more than 15% to be at high risk of bias for this domain (13, 16, 18). We considered four studies to be at low risk of attrition bias as attrition was much lower (14, 15, 19, 20). We judged the remaining study to be at unclear risk of attrition as it did not report on attrition (23).

### Selective Reporting

All nine studies were rated as unclear risk of reporting bias since no protocol is available to be compared to the respective published reports to identify any unreported outcomes (13–20, 23).

### Effects of Interventions

#### Primary Outcome—Treatment Failure

##### Oral Acyclovir vs. Placebo

**Figure 4:**
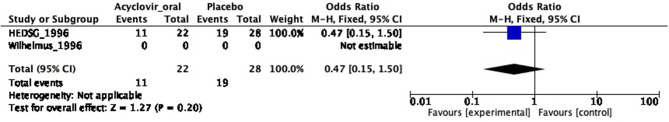
Forest plot comparing treatment failure in herpes simplex virus anterior uveitis patients treated with oral acyclovir vs. placebo.

**Figure 5:**
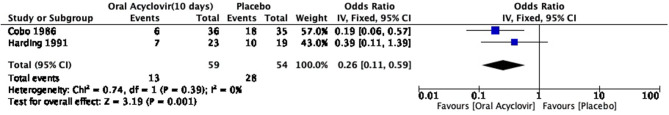
Forest plot comparing treatment failure in herpes zoster ophthalmicus patients developing anterior uveitis treated with oral acyclovir vs. placebo.

Two studies reported on the number of treatment failure in HSV anterior uveitis patients treated with oral acyclovir (10 week course) vs. placebo (20, 23). The forest plot (Figure 4) does not show any significant superiority of oral acyclovir over placebo (OR 0.47, 95% CI 0.15–1.50). Two other studies reported on the number of treatment failure in herpes zoster ophthalmicus (HZO) patients developing anterior uveitis treated with oral acyclovir (10 day course) vs. placebo (13, 15). The forest plot (Figure 5) shows significant superiority of oral acyclovir over placebo (OR 0.26, 95% CI 0.11–0.59).

##### Oral Acyclovir 7 vs. 14 Day Course

One study reported on the number of treatment failures in HZO patients developing anterior uveitis treated with oral acyclovir 7 day course vs. 14 day course (16). The forest plot does not show any significant superiority of one duration of treatment over the other (OR 0.21, 95% CI 0.01–4.50) (Table 3).

**Table 3:** Results of comparing treatment failure in herpes zoster ophthalmicus patients treated with Oral Acyclovir 7 vs. 14 Day Course, Oral Acyclovir vs. Oral Valaciclovir, and Topical Acyclovir vs. Oral Acyclovir.

**Study**	**Odds Ratio (IV, Fixed, 95% CI)**
Hoang-Xuan et al. (16)	0.21 (0.01, 4.50)
Colin et al. (14)	1.40 (0.48, 4.07)
Neoh et al. (17)	4.17 (1.28, 13.52)

##### Oral Acyclovir vs. Oral Valaciclovir

One study reported on the number of treatment failures in HZO patients developing anterior uveitis treated with oral acyclovir (7 day course) vs. oral valaciclovir (14). The forest plot does not show any significant superiority of oral acyclovir over oral valaciclovir (OR 1.40, 95% CI 0.48–4.07) (Table 3).

##### Topical Acyclovir vs. Oral Acyclovir

One study reported on the number of treatment failures in HZO patients developing anterior uveitis treated with topical acyclovir vs. oral acyclovir (7 day course) (17). The forest plot does show significant superiority of oral acyclovir over topical acyclovir (OR 4.17, 95% CI 1.28–13.52) (Table 3).

##### Topical Acyclovir vs. Topical Corticosteroids

**Figure 6:**
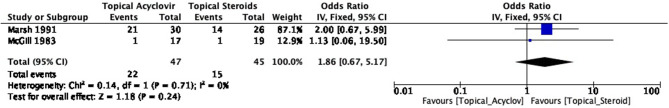
Forest plot comparing treatment failure in varicella zoster virus anterior uveitis patients treated with topical acyclovir vs. topical corticosteroids.

Two studies reported on the number of treatment failures in VZV anterior uveitis patients treated with topical acyclovir vs. topical corticosteroids (18, 19). The forest plot (Figure 6) does not show any significant superiority of topical acyclovir over topical corticosteroids (OR 1.86, 95% CI 0.67–5.17).

One study analyzed the rate of recurrence of HSV anterior uveitis among patients treated with oral acyclovir vs. placebo (33.3 and 22.2%, respectively) (20), whereas, two studies reported on patients with VZV anterior uveitis experiencing recurrences after treatment with topical acyclovir vs. topical corticosteroids (from 0 to 18.7%, and from 31.8 to 63%, respectively) (18, 19).

Results related to adverse effects of systemic antiviral treatment regimen are available for oral acyclovir only and analyzed in four studies (14–16, 20). 4.5–52.1% of patients experienced side effects (15, 20). Recurrences of uveitis attacks and adverse events to systemic antiviral medications are described in Table 4.

**Table 4:** Recurrences and Adverse reactions.

**S/N**	**References**	**Recurrences**	**Adverse reactions**
**1**	The Herpetic Eye Disease Study Group (20)	33.3% in oral acyclovir treated group; 22.2% in placebo treated group	4.5% in oral acyclovir treated group; 0% in placebo treated group
**2**	Wilhelmus etal. (23)	Not reported	Not reported
**3**	Marsh and Cooper (18)	18.7% in the topical acyclovir treated group; 31.8% in topical corticosteroids treated group; 34.6% in topical acyclovir and topical corticosteroids treated group	Not reported
**4**	McGill and Chapmen (19)	0% in topical acyclovir treated group; 63% in topical corticosteroids treated group	Not reported
**5**	Cobo et al. (13)	Not reported	Not reported
**6**	Colin et al. (14)	Not reported	14.8% in oral acyclovir treated group; 0% in oral valaciclovir treated group
**7**	Harding and Porter (15)	Not reported	52.1% in oral acyclovir treated group; 0% in placebo group
**8**	Hoang-Xuan et al. (16)	Not reported	17.1% in 7 day course oral acyclovir group; 13.3% in 14 day course group
**9**	Neoh et al. (17)	Not reported	Not reported

## Discussion

This study analyzed the occurrence of treatment failure in HSV and VZV anterior uveitis and ocular complications in herpes zoster ophthalmicus in patients treated with different antiviral therapies, administered alone or in combination with other agents, including corticosteroids. The review included nine randomized controlled trials (RCTs). Systematic search of scientific literature revealed that only two studies were specifically tailored to analyse whether the well-established and widely used systemic treatment approach with acyclovir was effective in HSV anterior uveitis (20, 23). Only two studies reported on the evidence for the use of topical acyclovir in VZV ocular inflammation (18, 19); whereas, five studies analyzed the effectiveness of systemic or topical acyclovir in preventing ocular complications from herpes zoster ophthalmicus (13, 15–17, 22). In addition, a significant research gap has been identified, as no data have been published since 2000. In particular, our forest plots (analysis 1–3) were constructed on old studies, the most recent of which was dated 1996. Although VAU is a relatively common condition, the small number of patients recruited suggested the impracticability of conducting larger studies.

We found that oral acyclovir was significantly effective over placebo in the treatment of VZV anterior uveitis patients. However, we could not derive the same conclusion for the treatment of HSV anterior uveitis. In addition, we could not establish any significant superiority of one treatment over the other in the management of VZV anterior uveitis and herpes zoster ophthalmicus, including topical acyclovir compared to oral acyclovir or topical corticosteroids, and oral acyclovir compared to a different length of treatment or oral valaciclovir.

Several outcome measures including those on recurrences, duration of therapy, choice of antiviral therapies, and topical/systemic routes of administration could not be assessed since there is lack of data in the published literature. Hence, only narrative review could be performed for these data points. Therefore, currently, the treatment of HSV and VZV anterior uveitis may be based on individual experiences and limited literature, largely due to weak clinical trial literature in this regard. Since VZV anterior uveitis is traditionally regarded to be more severe, antiviral therapy is routinely recommended in addition to anti-inflammatory therapy (28). In HSV anterior uveitis, further research is needed in clearly defining the role of antiviral therapy as observed from our meta-analysis. As there were no more than two RCTs included in the study to compare the same treatment approach, the efficacy of one treatment over another could not be conclusively established. In addition, even though effort has been made in the past to assess and compare different treatment approaches, our study identified a huge research gap, since no data have been published over the last 20 years. The results of this study will guide the research questions of future. We would hope that such trials will address the research gap, and rigorously evaluate the efficacy of different antiviral therapies in the treatment of VAU.

Limitations of the study include selection, performance, detection and reporting bias, with most study defined at unclear risk of bias. There was also a lack of data available in the full text from some of the studies included, such as details of the drugs administered, and their duration. Due to these factors, there is a lot of confusion in the ophthalmic community regarding the type of antiviral drug and the duration of antiviral and corticosteroid therapy that is optimum for patients diagnosed with VAU. There is a need for prospective studies to establish evidence-based guidelines for the management of this condition. Further, there is limited data on the management of complications of VAU such as glaucoma, cataract and other issues such as the role of PCR. None of the patients in the enrolled studies had confirmation of the viral agent using PCR from ocular fluids. In the current era, PCR detection of the type of viral agent is considered to be the standard of care. This represents a major limitation in the analysis.

In conclusion, from our research, it emerged that the current management of VAU is based on weak evidence and robust clinical data especially on therapeutics is needed. As a result, the ophthalmic fraternity is still unable to ask the question: how best do I manage a patient with VAU? In this regard, a multicentre approach would be beneficial in establishing treatment guidelines. Our study group has initiated a multicentre collaborative effort—The Infectious Uveitis Treatment Algorithm Network (TITAN)—aimed at establishing evidence and experience based guidelines for the management of patients with intraocular infectious diseases, including VAU. The collaborative efforts will look at establishing treatment algorithms for the management of anterior uveitis secondary to HSV and VZV through extensive literature review and collation of uveitis expert opinions from around the world. It is our hope these guidelines will serve as an important resource for clinicians managing patients with VAU.

## Data Availability Statement

The original contributions presented in the study are included in the article/Supplementary Material, further inquiries can be directed to the corresponding author.

## Author Contributions

VG, CP, RA, MW, and S-PC contributed to conception and design of the study. NJ, ND, AAh, MD, and AK performed the statistical analysis. IT and KA wrote the first draft of the manuscript. AAg, ZT, and SL wrote sections of the manuscript. All authors contributed to manuscript revision, read, and approved the submitted version.

## Conflict of Interest

The authors declare that the research was conducted in the absence of any commercial or financial relationships that could be construed as a potential conflict of interest.
