# Supplementary material for: Antiviral Therapy for Varicella Zoster Virus (VZV) and Herpes Simplex Virus (HSV)-Induced Anterior Uveitis: A Systematic Review and Meta-Analysis
Source: Front Med (Lausanne). 2021 Jul 2;8:686427. doi: 10.3389/fmed.2021.686427 (PMC8284188; doi:10.3389/fmed.2021.686427)
Supplement: Supplementary file 2 [file Data_Sheet_2.DOCX]

**History and Search Details**

Download

Delete

| **Search** | **Actions** | **Details** | **Query** | **Results** | **Time** |
| --- | --- | --- | --- | --- | --- |
| #38 |  |  | Search: **(((((((((("Uveitis, Anterior"[Mesh]) OR (Anterior Uveitides OR Anterior Uveitis OR Uveitides, Anterior)) OR (Herpes Simplex virus)) OR (HSV)) OR ("Simplexvirus"[Mesh])) OR (Simplexviruses OR Herpes Simplex Virus OR Herpes Simplex Viruses OR Herpesvirus Hominis OR Herpesvirus Homini OR Homini, Herpesvirus OR Hominis, Herpesvirus OR Herpesvirus 1, Saimiriine OR Saimiriine Herpesvirus 1 OR Herpesvirus 1, Saimirine OR Saimirine Herpesvirus 1 OR Herpesvirus 1 (alpha), Saimirine OR Herpesvirus Platyrhinae OR Platyrhinae, Herpesvirus)) OR (Varicella Zoster virus)) OR ("Varicella Zoster Virus Infection"[Mesh])) OR (Congenital Varicella Syndrome)) AND (((((((((((((((acyclovir) OR ("Acyclovir"[Mesh])) OR ("9-2-Hydroxyethoxymethyl guanine" OR Aciclovir OR Acycloguanosine)) OR (valacyclovir)) OR ("Valacyclovir"[Mesh])) OR (Valaciclovir OR BW256U87 OR Valacyclovir, X-Hydrochloride, DL-isomer OR Valacyclovir Hydrochloride, DL-isomer OR Valacyclovir, D-isomer OR Valacyclovir, D OR D- Valacyclovir OR Valacyclovir, L-isomer OR L-Valylacyclovir OR L Valylacyclovir OR Acyclovir, L-valyl Ester OR Acyclovir, L valyl Ester OR L-valyl Ester Acyclovir OR Valacyclovir, X-Hydrochloride, D-isomer OR Valacyclovir Hydrochloride256U87 OR Valacyclovir, DL-isomer)) OR (famciclovir)) OR ("Famciclovir"[Mesh])) OR (9-(4-Acetoxy-3-(acetoxymethyl)but-1-yl)-2-aminopurine OR 1,3-Propanediol, 2-(2-(2-amino-9H-purin-9-yl)ethyl)-, diacetate (ester) OR BRL 42810 OR BRL-42810 OR BRL42810 OR Famvir)) OR (ganciclovir)) OR ("Ganciclovir"[Mesh])) OR (Gancyclovir OR BW-759 OR Ganciclovir Sodium OR Ganciclovir, Monosodium Salt OR RS-21592 OR BIOLF-62 OR Cytovene)) OR (valganciclovir)) OR ("Valganciclovir"[Mesh])) OR (Ganciclovir L-valyl Ester OR Ganciclovir L valyl Ester OR Valcyt OR Valganciclovir Hydrochloride OR Valcyte))) AND ((randomized controlled trial) OR (non- randomized controlled trials))** | [503](https://pubmed.ncbi.nlm.nih.gov/?term=longqueryc00493adfac65d92f0b7&sort=relevance&size=100&long_term_hash=longqueryc00493adfac65d92f0b7) | 06:31:39 |
| #37 |  |  | Search: **(randomized controlled trial) OR (non- randomized controlled trials)** | [678,638](https://pubmed.ncbi.nlm.nih.gov/?term=%28randomized+controlled+trial%29+OR+%28non-+randomized+controlled+trials%29&sort=relevance&size=100) | 06:31:25 |
| #36 |  |  | Search: **non- randomized controlled trials** | [85,777](https://pubmed.ncbi.nlm.nih.gov/?term=non-+randomized+controlled+trials&size=100&sort=relevance) | 06:31:13 |
| #35 |  |  | Search: **randomized controlled trial** | [670,587](https://pubmed.ncbi.nlm.nih.gov/?term=randomized+controlled+trial&size=100&sort=relevance) | 06:30:49 |
| #34 |  |  | Search: **((((((((("Uveitis, Anterior"[Mesh]) OR (Anterior Uveitides OR Anterior Uveitis OR Uveitides, Anterior)) OR (Herpes Simplex virus)) OR (HSV)) OR ("Simplexvirus"[Mesh])) OR (Simplexviruses OR Herpes Simplex Virus OR Herpes Simplex Viruses OR Herpesvirus Hominis OR Herpesvirus Homini OR Homini, Herpesvirus OR Hominis, Herpesvirus OR Herpesvirus 1, Saimiriine OR Saimiriine Herpesvirus 1 OR Herpesvirus 1, Saimirine OR Saimirine Herpesvirus 1 OR Herpesvirus 1 (alpha), Saimirine OR Herpesvirus Platyrhinae OR Platyrhinae, Herpesvirus)) OR (Varicella Zoster virus)) OR ("Varicella Zoster Virus Infection"[Mesh])) OR (Congenital Varicella Syndrome)) AND (((((((((((((((acyclovir) OR ("Acyclovir"[Mesh])) OR ("9-2-Hydroxyethoxymethyl guanine" OR Aciclovir OR Acycloguanosine)) OR (valacyclovir)) OR ("Valacyclovir"[Mesh])) OR (Valaciclovir OR BW256U87 OR Valacyclovir, X-Hydrochloride, DL-isomer OR Valacyclovir Hydrochloride, DL-isomer OR Valacyclovir, D-isomer OR Valacyclovir, D OR D- Valacyclovir OR Valacyclovir, L-isomer OR L-Valylacyclovir OR L Valylacyclovir OR Acyclovir, L-valyl Ester OR Acyclovir, L valyl Ester OR L-valyl Ester Acyclovir OR Valacyclovir, X-Hydrochloride, D-isomer OR Valacyclovir Hydrochloride256U87 OR Valacyclovir, DL-isomer)) OR (famciclovir)) OR ("Famciclovir"[Mesh])) OR (9-(4-Acetoxy-3-(acetoxymethyl)but-1-yl)-2-aminopurine OR 1,3-Propanediol, 2-(2-(2-amino-9H-purin-9-yl)ethyl)-, diacetate (ester) OR BRL 42810 OR BRL-42810 OR BRL42810 OR Famvir)) OR (ganciclovir)) OR ("Ganciclovir"[Mesh])) OR (Gancyclovir OR BW-759 OR Ganciclovir Sodium OR Ganciclovir, Monosodium Salt OR RS-21592 OR BIOLF-62 OR Cytovene)) OR (valganciclovir)) OR ("Valganciclovir"[Mesh])) OR (Ganciclovir L-valyl Ester OR Ganciclovir L valyl Ester OR Valcyt OR Valganciclovir Hydrochloride OR Valcyte))** | [9,514](https://pubmed.ncbi.nlm.nih.gov/?term=longquery0aeaf7cd9d6d7ab7f786&sort=relevance&size=100&long_term_hash=longquery0aeaf7cd9d6d7ab7f786) | 06:30:17 |
| #33 |  |  | Search: **((((((((((((((acyclovir) OR ("Acyclovir"[Mesh])) OR ("9-2-Hydroxyethoxymethyl guanine" OR Aciclovir OR Acycloguanosine)) OR (valacyclovir)) OR ("Valacyclovir"[Mesh])) OR (Valaciclovir OR BW256U87 OR Valacyclovir, X-Hydrochloride, DL-isomer OR Valacyclovir Hydrochloride, DL-isomer OR Valacyclovir, D-isomer OR Valacyclovir, D OR D- Valacyclovir OR Valacyclovir, L-isomer OR L-Valylacyclovir OR L Valylacyclovir OR Acyclovir, L-valyl Ester OR Acyclovir, L valyl Ester OR L-valyl Ester Acyclovir OR Valacyclovir, X-Hydrochloride, D-isomer OR Valacyclovir Hydrochloride256U87 OR Valacyclovir, DL-isomer)) OR (famciclovir)) OR ("Famciclovir"[Mesh])) OR (9-(4-Acetoxy-3-(acetoxymethyl)but-1-yl)-2-aminopurine OR 1,3-Propanediol, 2-(2-(2-amino-9H-purin-9-yl)ethyl)-, diacetate (ester) OR BRL 42810 OR BRL-42810 OR BRL42810 OR Famvir)) OR (ganciclovir)) OR ("Ganciclovir"[Mesh])) OR (Gancyclovir OR BW-759 OR Ganciclovir Sodium OR Ganciclovir, Monosodium Salt OR RS-21592 OR BIOLF-62 OR Cytovene)) OR (valganciclovir)) OR ("Valganciclovir"[Mesh])) OR (Ganciclovir L-valyl Ester OR Ganciclovir L valyl Ester OR Valcyt OR Valganciclovir Hydrochloride OR Valcyte)** | [20,812](https://pubmed.ncbi.nlm.nih.gov/?term=%28%28%28%28%28%28%28%28%28%28%28%28%28%28acyclovir%29+OR+%28%22Acyclovir%22%5BMesh%5D%29%29+OR+%28%229-2-Hydroxyethoxymethyl+guanine%22+OR+Aciclovir+OR+Acycloguanosine%29%29+OR+%28valacyclovir%29%29+OR+%28%22Valacyclovir%22%5BMesh%5D%29%29+OR+%28Valaciclovir+OR+BW256U87+OR+Valacyclovir%2C+X-Hydrochloride%2C+DL-isomer+OR+Valacyclovir+Hydrochloride%2C+DL-isomer+OR+Valacyclovir%2C+D-isomer+OR+Valacyclovir%2C+D+OR+D-+Valacyclovir+OR+Valacyclovir%2C+L-isomer+OR+L-Valylacyclovir+OR+L+Valylacyclovir+OR+Acyclovir%2C+L-valyl+Ester+OR+Acyclovir%2C+L+valyl+Ester+OR+L-valyl+Ester+Acyclovir+OR+Valacyclovir%2C+X-Hydrochloride%2C+D-isomer+OR+Valacyclovir+Hydrochloride256U87+OR+Valacyclovir%2C+DL-isomer%29%29+OR+%28famciclovir%29%29+OR+%28%22Famciclovir%22%5BMesh%5D%29%29+OR+%289-%284-Acetoxy-3-%28acetoxymethyl%29but-1-yl%29-2-aminopurine+OR+1%2C3-Propanediol%2C+2-%282-%282-amino-9H-purin-9-yl%29ethyl%29-%2C+diacetate+%28ester%29+OR+BRL+42810+OR+BRL-42810+OR+BRL42810+OR+Famvir%29%29+OR+%28ganciclovir%29%29+OR+%28%22Ganciclovir%22%5BMesh%5D%29%29+OR+%28Gancyclovir+OR+BW-759+OR+Ganciclovir+Sodium+OR+Ganciclovir%2C+Monosodium+Salt+OR+RS-21592+OR+BIOLF-62+OR+Cytovene%29%29+OR+%28valganciclovir%29%29+OR+%28%22Valganciclovir%22%5BMesh%5D%29%29+OR+%28Ganciclovir+L-valyl+Ester+OR+Ganciclovir+L+valyl+Ester+OR+Valcyt+OR+Valganciclovir+Hydrochloride+OR+Valcyte%29&sort=relevance&size=100) | 06:29:49 |
| #32 |  |  | Search: **Ganciclovir L-valyl Ester OR Ganciclovir L valyl Ester OR Valcyt OR Valganciclovir Hydrochloride OR Valcyte** | [1,335](https://pubmed.ncbi.nlm.nih.gov/?term=Ganciclovir+L-valyl+Ester+OR+Ganciclovir+L+valyl+Ester+OR+Valcyt+OR+Valganciclovir+Hydrochloride+OR+Valcyte&size=100&sort=relevance) | 06:28:53 |
| #31 |  |  | Search: **"Valganciclovir"[Mesh]** Sort by: **Most Recent** | [767](https://pubmed.ncbi.nlm.nih.gov/?sort=date&term=%22Valganciclovir%22%5BMesh%5D&size=100) | 06:28:28 |
| #30 |  |  | Search: **valganciclovir** | [1,333](https://pubmed.ncbi.nlm.nih.gov/?term=valganciclovir&size=100&sort=relevance) | 06:28:01 |
| #29 |  |  | Search: **Gancyclovir OR BW-759 OR Ganciclovir Sodium OR Ganciclovir, Monosodium Salt OR RS-21592 OR BIOLF-62 OR Cytovene** | [8,971](https://pubmed.ncbi.nlm.nih.gov/?term=Gancyclovir+OR+BW-759+OR+Ganciclovir+Sodium+OR+Ganciclovir%2C+Monosodium+Salt+OR+RS-21592+OR+BIOLF-62+OR+Cytovene&size=100&sort=relevance) | 06:27:40 |
| #28 |  |  | Search: **"Ganciclovir"[Mesh]** Sort by: **Most Recent** | [6,180](https://pubmed.ncbi.nlm.nih.gov/?sort=date&term=%22Ganciclovir%22%5BMesh%5D&size=100) | 06:27:17 |
| #27 |  |  | Search: **ganciclovir** | [8,803](https://pubmed.ncbi.nlm.nih.gov/?term=ganciclovir&size=100&sort=relevance) | 06:26:43 |
| #26 |  |  | Search: **9-(4-Acetoxy-3-(acetoxymethyl)but-1-yl)-2-aminopurine OR 1,3-Propanediol, 2-(2-(2-amino-9H-purin-9-yl)ethyl)-, diacetate (ester) OR BRL 42810 OR BRL-42810 OR BRL42810 OR Famvir** | [836](https://pubmed.ncbi.nlm.nih.gov/?term=9-%284-Acetoxy-3-%28acetoxymethyl%29but-1-yl%29-2-aminopurine+OR+1%2C3-Propanediol%2C+2-%282-%282-amino-9H-purin-9-yl%29ethyl%29-%2C+diacetate+%28ester%29+OR+BRL+42810+OR+BRL-42810+OR+BRL42810+OR+Famvir&size=100&sort=relevance) | 06:26:21 |
| #25 |  |  | Search: **"Famciclovir"[Mesh]** Sort by: **Most Recent** | [530](https://pubmed.ncbi.nlm.nih.gov/?sort=date&term=%22Famciclovir%22%5BMesh%5D&size=100) | 06:25:55 |
| #24 |  |  | Search: **famciclovir** | [833](https://pubmed.ncbi.nlm.nih.gov/?term=famciclovir&size=100&sort=relevance) | 06:24:54 |
| #23 |  |  | Search: **Valaciclovir OR BW256U87 OR Valacyclovir, X-Hydrochloride, DL-isomer OR Valacyclovir Hydrochloride, DL-isomer OR Valacyclovir, D-isomer OR Valacyclovir, D OR D- Valacyclovir OR Valacyclovir, L-isomer OR L-Valylacyclovir OR L Valylacyclovir OR Acyclovir, L-valyl Ester OR Acyclovir, L valyl Ester OR L-valyl Ester Acyclovir OR Valacyclovir, X-Hydrochloride, D-isomer OR Valacyclovir Hydrochloride256U87 OR Valacyclovir, DL-isomer** | [1,649](https://pubmed.ncbi.nlm.nih.gov/?term=Valaciclovir+OR+BW256U87+OR+Valacyclovir%2C+X-Hydrochloride%2C+DL-isomer+OR+Valacyclovir+Hydrochloride%2C+DL-isomer+OR+Valacyclovir%2C+D-isomer+OR+Valacyclovir%2C+D+OR+D-+Valacyclovir+OR+Valacyclovir%2C+L-isomer+OR+L-Valylacyclovir+OR+L+Valylacyclovir+OR+Acyclovir%2C+L-valyl+Ester+OR+Acyclovir%2C+L+valyl+Ester+OR+L-valyl+Ester+Acyclovir+OR+Valacyclovir%2C+X-Hydrochloride%2C+D-isomer+OR+Valacyclovir+Hydrochloride256U87+OR+Valacyclovir%2C+DL-isomer&size=100&sort=relevance) | 06:23:49 |
| #16 |  |  | Search: **"Valacyclovir"[Mesh]** Sort by: **Most Recent** | [1,066](https://pubmed.ncbi.nlm.nih.gov/?sort=date&term=%22Valacyclovir%22%5BMesh%5D&size=100) | 06:15:08 |
| #15 |  |  | Search: **valacyclovir** | [1,470](https://pubmed.ncbi.nlm.nih.gov/?term=valacyclovir&size=100&sort=relevance) | 06:14:32 |
| #14 |  |  | Search: **"9-2-Hydroxyethoxymethyl guanine" OR Aciclovir OR Acycloguanosine** | [17,630](https://pubmed.ncbi.nlm.nih.gov/?term=%229-2-Hydroxyethoxymethyl+guanine%22+OR+Aciclovir+OR+Acycloguanosine&size=100&sort=relevance) | 06:14:10 |
| #13 |  |  | Search: **"Acyclovir"[Mesh]** Sort by: **Most Recent** | [14,253](https://pubmed.ncbi.nlm.nih.gov/?sort=date&term=%22Acyclovir%22%5BMesh%5D&size=100) | 06:13:05 |
| #12 |  |  | Search: **acyclovir** | [17,554](https://pubmed.ncbi.nlm.nih.gov/?term=acyclovir++&size=100&sort=relevance) | 06:12:24 |
| #11 |  |  | Search: **(((((((("Uveitis, Anterior"[Mesh]) OR (Anterior Uveitides OR Anterior Uveitis OR Uveitides, Anterior)) OR (Herpes Simplex virus)) OR (HSV)) OR ("Simplexvirus"[Mesh])) OR (Simplexviruses OR Herpes Simplex Virus OR Herpes Simplex Viruses OR Herpesvirus Hominis OR Herpesvirus Homini OR Homini, Herpesvirus OR Hominis, Herpesvirus OR Herpesvirus 1, Saimiriine OR Saimiriine Herpesvirus 1 OR Herpesvirus 1, Saimirine OR Saimirine Herpesvirus 1 OR Herpesvirus 1 (alpha), Saimirine OR Herpesvirus Platyrhinae OR Platyrhinae, Herpesvirus)) OR (Varicella Zoster virus)) OR ("Varicella Zoster Virus Infection"[Mesh])) OR (Congenital Varicella Syndrome)** | [85,020](https://pubmed.ncbi.nlm.nih.gov/?term=%28%28%28%28%28%28%28%28%22Uveitis%2C+Anterior%22%5BMesh%5D%29+OR+%28Anterior+Uveitides+OR+Anterior+Uveitis+OR+Uveitides%2C+Anterior%29%29+OR+%28Herpes+Simplex+virus%29%29+OR+%28HSV%29%29+OR+%28%22Simplexvirus%22%5BMesh%5D%29%29+OR+%28Simplexviruses+OR+Herpes+Simplex+Virus+OR+Herpes+Simplex+Viruses+OR+Herpesvirus+Hominis+OR+Herpesvirus+Homini+OR+Homini%2C+Herpesvirus+OR+Hominis%2C+Herpesvirus+OR+Herpesvirus+1%2C+Saimiriine+OR+Saimiriine+Herpesvirus+1+OR+Herpesvirus+1%2C+Saimirine+OR+Saimirine+Herpesvirus+1+OR+Herpesvirus+1+%28alpha%29%2C+Saimirine+OR+Herpesvirus+Platyrhinae+OR+Platyrhinae%2C+Herpesvirus%29%29+OR+%28Varicella+Zoster+virus%29%29+OR+%28%22Varicella+Zoster+Virus+Infection%22%5BMesh%5D%29%29+OR+%28Congenital+Varicella+Syndrome%29&sort=relevance&size=100) | 06:11:42 |
| #10 |  |  | Search: **Congenital Varicella Syndrome** | [19,977](https://pubmed.ncbi.nlm.nih.gov/?term=Congenital+Varicella+Syndrome&size=100&sort=relevance) | 06:09:46 |
| #9 |  |  | Search: **"Varicella Zoster Virus Infection"[Mesh]** Sort by: **Most Recent** | [18,085](https://pubmed.ncbi.nlm.nih.gov/?sort=date&term=%22Varicella+Zoster+Virus+Infection%22%5BMesh%5D&size=100) | 06:09:29 |
| #8 |  |  | Search: **Varicella Zoster virus** | [11,362](https://pubmed.ncbi.nlm.nih.gov/?term=Varicella+Zoster+virus&size=100&sort=relevance) | 06:08:38 |
| #7 |  |  | Search: **"Simplexvirus"[Mesh]** Sort by: **Most Recent** | [30,437](https://pubmed.ncbi.nlm.nih.gov/?sort=date&term=%22Simplexvirus%22%5BMesh%5D&size=100) | 06:08:08 |
| #6 |  |  | Search: **Anterior Uveitides OR Anterior Uveitis OR Uveitides, Anterior** | [17,774](https://pubmed.ncbi.nlm.nih.gov/?term=Anterior+Uveitides+OR+Anterior+Uveitis+OR+Uveitides%2C+Anterior&size=100&sort=relevance) | 06:06:48 |
| #5 |  |  | Search: **"Uveitis, Anterior"[Mesh]** Sort by: **Most Recent** | [14,151](https://pubmed.ncbi.nlm.nih.gov/?sort=date&term=%22Uveitis%2C+Anterior%22%5BMesh%5D&size=100) | 06:05:49 |
| #4 |  |  | Search: **Viral anterior uveitis** | [649](https://pubmed.ncbi.nlm.nih.gov/?term=Viral+anterior+uveitis&size=100&sort=relevance) | 06:04:00 |
| #3 |  |  | Search: **Simplexviruses OR Herpes Simplex Virus OR Herpes Simplex Viruses OR Herpesvirus Hominis OR Herpesvirus Homini OR Homini, Herpesvirus OR Hominis, Herpesvirus OR Herpesvirus 1, Saimiriine OR Saimiriine Herpesvirus 1 OR Herpesvirus 1, Saimirine OR Saimirine Herpesvirus 1 OR Herpesvirus 1 (alpha), Saimirine OR Herpesvirus Platyrhinae OR Platyrhinae, Herpesvirus** | [45,609](https://pubmed.ncbi.nlm.nih.gov/?term=Simplexviruses+OR+Herpes+Simplex+Virus+OR+Herpes+Simplex+Viruses+OR+Herpesvirus+Hominis+OR+Herpesvirus+Homini+OR+Homini%2C+Herpesvirus+OR+Hominis%2C+Herpesvirus+OR+Herpesvirus+1%2C+Saimiriine+OR+Saimiriine+Herpesvirus+1+OR+Herpesvirus+1%2C+Saimirine+OR+Saimirine+Herpesvirus+1+OR+Herpesvirus+1+%28alpha%29%2C+Saimirine+OR+Herpesvirus+Platyrhinae+OR+Platyrhinae%2C+Herpesvirus&size=100&sort=relevance) | 06:00:32 |
| #2 |  |  | Search: **HSV** | [24,766](https://pubmed.ncbi.nlm.nih.gov/?term=HSV&size=100&sort=relevance) | 05:59:55 |
| #1 |  |  | Search: **Herpes Simplex virus** | [45,248](https://pubmed.ncbi.nlm.nih.gov/?term=Herpes+Simplex+virus+&size=100&sort=relevance) | 05:59:30 |

Simplexviruses OR "Herpes Simplex Virus" OR "Herpes Simplex Viruses" OR "Herpesvirus Hominis" OR "Herpesvirus Homini" OR "Homini, Herpesvirus" OR "Hominis, Herpesvirus" OR "Herpesvirus 1, Saimiriine" OR "Saimiriine Herpesvirus 1" OR "Herpesvirus 1, Saimirine" OR "Saimirine Herpesvirus 1" OR "Herpesvirus 1 (alpha), Saimirine" OR "Herpesvirus Platyrhinae" OR "Platyrhinae, Herpesvirus"

**"Uveitis, Anterior"OR “Anterior Uveitides” OR “Anterior Uveitis” OR “Uveitides, Anterior” OR “Herpes Simplex virus” OR HSV OR "Simplexvirus" OR Simplexviruses OR “Herpes Simplex Viruses” OR “Herpesvirus Hominis” OR “Herpesvirus Homini” OR Homini, Herpesvirus OR Hominis, Herpesvirus OR Herpesvirus 1, Saimiriine OR Saimiriine Herpesvirus 1 OR Herpesvirus 1, Saimirine OR Saimirine Herpesvirus 1 OR Herpesvirus 1 (alpha), Saimirine OR Herpesvirus Platyrhinae OR Platyrhinae, Herpesvirus)) OR (Varicella Zoster virus)) OR ("Varicella Zoster Virus Infection"[Mesh])) OR (Congenital Varicella Syndrome)**

Simplexviruses OR "Herpes Simplex Viruses" OR "Herpesvirus Hominis" OR "Herpesvirus Homini" OR "Homini, Herpesvirus" OR "Hominis, Herpesvirus" OR "Herpesvirus 1, Saimiriine" OR "Saimiriine Herpesvirus 1" OR "Herpesvirus 1, Saimirine" OR "Saimirine Herpesvirus 1" OR "Herpesvirus 1 (alpha), Saimirine" OR "Herpesvirus Platyrhinae" OR "Platyrhinae, Herpesvirus"
